# Diseases of the Urinary Organs

**Published:** 1873-05

**Authors:** 


*Diseases of the Urinary Organs.* By John W. S. Gouley, M. D. New York: Wm. Wood & Co. Buffalo: H. H. Otis.

Any new help that may be afforded in the management of the surgical diseases of which this work treats, will be received no doubt, with welcome Our author's extended experience with these diseases, both in public and private practice, eminently qualifies him to speak with authority, while his methods commend themselves by their practical ingenuity and efficiency.

The book, which deals only with the male subject, has the happy character of clinical observation and teaching. A point of especial interest to practitioners in general, is that of the catheterism of strictures, and to this Dr. Gouley has given much attention. He says: —

Granular urethritis, in accordance with the views of M. Desormeaux, which I believe to be correct in the main, is the formative stage of stricture. This chronic inflammation, if allowed to progress, ends by destroying the mucous glands, and the membrane loses its identity; its epithelial investment soon ceases to afford any protection against the irritating urine, as most of the cells are cast off before they are fully developed, and pass away as pus corpuscles. In the next stage, there is cell-proliferation in the meshes of the mucous membrane, and also in the sub-mucous connective tissue. In the third stage the new cells are transformed into fibrils, after which retraction takes place as in the tissue of cicatrices, and then the true organic or inodular stricture is developed.

In the treatment of the formative stage he uses dilatation with bougies and sounds; and if after six weeks or two months of treatment by dilatation and expansion the discharge still persists, the obstinate granular urethritis is treated on the same general plan as granular lids — with mitigated nitrate of silver, weak solution of sulphate of copper, &c. For penetrating strictures difficult of passage, the author has devised and made use of capillary probe-pointed bougies of whalebone, which can by manipulation, be insinuated into the bladder, when over one of these, as a center, is easily passed a tunnelled sound by which dilatation is effected. If a single whalebone guide is hindered in its course, others are introduced by its side till the passage is penetrated. These means have been very successful in cases where the ordinary bougies fail of introduction.

In preference to urethrotomy, he always prefers, in strictures beyond the pendulous portion of the urethra, employing divulsion by sounds or by an instrument for the purpose. Warning against the use of undue violence to the urethra, and its liability to the causing of urethral fever of a violent and sometimes speedily fatal character, is strongly impressed upon the reader.

His remarks upon external perineal urethrotomy are extended and valuable. He says that the retention of a catheter in the bladder after the operation, even for forty-eight hours, is not only unnecessary, but harmful; that it does not fulfil the indications for which it is used, and is, as a general rule, attended with danger. He gives the history of thirteen successful cases in which no catheter was retained after urethrotomy for stricture, and as many similar after the same from traumatic lesions, though after complete traumatic division or occlusion, an exception to the rule may possibly be made.

In retention of urine and extravasation of the same, he recommends the attendant to make the initial incisions for urethrotomy at once, so as to allow drainage, and operation may follow as soon as convenient. In treating cases of immediate danger from retention, he adduces the conclusions of M. Labbe: — 1. That capillary hypogastric puncture is a perfectly harmless operation. 2. That in all cases it must be substituted for ordinary hypogastric puncture. 3. That in a great number of cases it may, when only once practised, allow the surgeon to penetrate afterwards into the bladder through the natural passages. 4. That in certain cases, where catheterism is impossible, it may be performed three or four times a day without any injurious effect, and thus permit the surgeon to gain time and restore the natural passages; and at the very least it constitutes a palliative means of the highest importance.

Respecting the removal of stone, he says the time will soon come when surgeons will take a special pride in showing detritus rather than specimens of large urinary calculi. Perineal lithotrity is the subject of the closing chapter, in which the operation, Dolbeau's instrument which dilates the prostrate, and the author's lithoclast, are described in detail.

The work is concise, clear, and will be examined with pleasure, by all those busied or interested in urethral and vesical surgery.

